# Integrating multi-omics and machine learning for disease resistance prediction in legumes

**DOI:** 10.1007/s00122-025-04948-2

**Published:** 2025-06-27

**Authors:** Shameela Mohamedikbal, Hawlader A. Al-Mamun, Mitchell S. Bestry, Jacqueline Batley, David Edwards

**Affiliations:** 1 Centre for Applied Bioinformatics, University of Western Australia, Perth, WA 6009 Australia; 2 School of Biological Sciences, University of Western Australia, Perth, WA 6009 Australia; 3 InterGrain Pty Ltd, Perth, WA 6163 Australia

## Abstract

**Key message:**

Multi-omics assisted prediction of disease resistance mechanisms using machine learning has the potential to accelerate the breeding of resistant legume varieties.

**Abstract:**

Grain legumes, such as soybean (*Glycine max* (L.) Merr.), chickpea (*Cicer arietinum* L.), and lentil (*Lens culinaris* Medik.) play an important role in combating micronutrient malnutrition in the growing human population. However, plant diseases significantly reduce grain yield, causing 10–40% losses in major food crops. The genetic mechanisms associated with disease resistance in legumes have been widely studied using genomic approaches. Multi-omics data encompassing various biological layers such as the transcriptome, epigenome, proteome, and metabolome, in addition to the genome, enables researchers to gain a deeper understanding of these complementary layers and their roles in complex legume-pathogen interactions. Genomic prediction, used to select the best genotypes with desirable traits for breeding, has largely relied on genome-wide markers and statistical approaches to estimate the breeding values of individuals. Integrating multi-omics data into genomic prediction can be achieved using machine learning models, which can capture nonlinear relationships prevalent in high-dimensional data better than traditional statistical methods. This integration may enable more accurate predictions and identification of resistance mechanisms for breeding resistant legumes. Despite its potential, multi-omics integration for disease resistance prediction in legumes has been largely unexplored. In this review, we explore omics studies focusing on disease resistance in legumes and discuss how machine learning models can integrate multi-omics data for disease resistance prediction. Such multi-omics assisted prediction has the potential to reduce the breeding cycle for developing disease-resistant legume varieties.

## Introduction

Micronutrient malnutrition, resulting from insufficient intake and absorption of essential vitamins and minerals, affects human health and contributes to higher maternal and child mortality rates (Black et al. 2013; Bailey et al. 2015; Kiani et al. 2022). Grain legumes, including soybean (*Glycine max* (L.) Merr.), chickpea (*Cicer arietinum* L.), and lentil (*Lens culinaris* Medik.) are particularly valuable in combating these deficiencies due to their rich micronutrient profiles (Graham and Vance 2003; Rehman et al. 2019). As the global population is expected to reach 9.8 billion by 2050, crop production must increase by 60–110% to meet dietary demands (Tilman et al. 2002; Alexandratos et al. 2012; Ray et al. 2013; United Nations Department of Economic and Social Affairs, Population Division 2022). However, the current annual growth rates of 1.1–1.7% for crops such as wheat (*Triticum aestivum* L.), rice (*Oryza sativa* L.) and soybean are insufficient to double crop production by 2050 (Iizumi et al. 2018). This goal is further complicated by plant diseases, which cause yield losses of 10 – 40% in major food crops (Strange and Scott 2005; Oerke 2006; Ristaino et al. 2021). Additionally, regions with fast-growing populations and existing food shortages experience the highest yield losses in major food crops due to disease (Savary et al. 2019). Interacting climatic variables, such as elevated temperatures, increased CO_2_ levels, and changing precipitation patterns, alter host resistance and facilitate pathogen evolution, further complicating the impact of disease on grain yield (Garrett et al. 2006; Singh et al. 2023a).

Plant disease management strategies, including crop rotation and chemical use, have been successful; however, deploying host resistance using resistant varieties remains the most effective method (Stuthman et al. 2007; Deng et al. 2020). Plants defend against pathogens using two main mechanisms: qualitative and quantitative resistance. Qualitative resistance is mediated by single major resistance (R) genes, while quantitative resistance is associated with multiple genes or quantitative trait loci (QTLs) (Flor 1971; St Clair 2010). Qualitative resistance provides near-complete protection against specific pathogenic strains but can break down due to continuous pathogen evolution (Leach et al. 2001). Quantitative resistance provides partial but durable resistance against various strains (Lynch and Walsh 1998; Niks et al. 2015). Next-generation sequencing (NGS) technologies have enabled the identification of quantitative resistance loci through QTL mapping and genome-wide association studies (GWAS) (Michael and Jackson 2013; Gangurde et al. 2022). Breeding varieties with strong resistance requires combining diverse loci from both quantitative and qualitative resistance without adversely affecting other desirable traits, such as yield, due to potential growth-defence trade-offs (Nelson et al. 2018; Derbyshire et al. 2024). This necessitates intervention through several biological pathways and mechanisms in resistance breeding, as disease resistance is influenced by multiple genes and environmental factors (Keen 1990; Poland et al. 2009; van Esse et al. 2020). Additionally, the narrow genetic base in self-pollinated legumes such as soybean limits sources of genetic variation for resistance breeding (Hyten et al. 2006).

In plant breeding, genomic selection (GS) enables the selection of plants with desirable traits using a set of genome-wide DNA markers, typically single-nucleotide polymorphisms (SNPs), to estimate the total genetic value or genomic estimated breeding values (GEBVs) of individuals in a breeding population (Meuwissen et al. 2001). This process relies on genomic prediction (GP), which uses statistical models trained on a well-characterised training population that shares some level of relatedness with the breeding population (Meuwissen et al. 2001). The training population consists of individuals with known genetic information and observed phenotypic traits (labelled data) (Meuwissen et al. 2001). The resulting GEBVs serve as pseudo-phenotypes that enable the selection of best parents without further phenotypic evaluations, thereby reducing breeding cycle lengths (Feng et al. 2023). GS therefore enhances the rate of annual genetic improvements in plant breeding, enabling faster development of varieties with desirable traits (Xu et al. 2017; Alemu et al. 2024). However, in the case of polygenic traits such as disease resistance, genomic data alone may not fully capture the dynamic changes in plant-pathogen interactions. Plant resistance to pathogens is also influenced by intermediate biological processes such as transcription, translation, and metabolic changes that occur in response to infection (Ritchie et al. 2015; Castro‑Moretti et al. 2020). To associate quantitative variation in phenotypes with the effects from different biological layers, researchers are adopting multi-omics approaches to study trait variation in plants (Cao et al. 2022; Roychowdhury et al. 2023). These approaches may enable more accurate predictions by integrating interactions between various biological layers into the models (Guo et al. 2016; Wu et al. 2022).

Multi-omics integrates data from various molecular levels, including DNA sequences (genomics), gene expression (transcriptomics), epigenetic modifications (epigenomics), protein (proteomics) and metabolite levels (metabolomics) (Yang et al. 2021). It provides a comprehensive understanding of the complementary biological levels associated with traits such as plant defence, extending beyond genetic markers to include protein and metabolite expression levels, and their role in phenotypic variability within genotypes (Kliebenstein 2012; Ritchie et al. 2015). For example, Shi et al. (2021) integrated transcriptomic and metabolomic data to identify candidate genes and potential metabolites in soybean varieties resistant to soybean cyst nematode infection. Moreover, omics has provided detailed insights into the molecular mechanisms behind growth, development, and stress responses in plants (Yang et al. 2021; Derbyshire et al. 2022).

A challenge in incorporating multi-omics data into GP pipelines lies in the heterogenous and often unstructured biological data generated from various omics layers (Picard et al. 2021). For instance, genomic data in the form of SNPs are encoded as numeric allele counts (0, 1, 2) for homozygous reference, homozygous alternate and heterozygous alleles, respectively. Transcriptomic data quantifies gene expression as raw counts, showing the number of RNA sequencing reads aligned to each gene in a reference genome (Wang et al. 2009; Finotello and Di Camillo 2015). These gene expression counts can vary from tens for lower-expressed genes to thousands for highly expressed genes. Such variation in the nature and distribution of multi-omics data poses challenges to traditional regression-based GP models that assume a linear relationship between genotype and phenotype (Pérez‑Rodríguez et al. 2012). Additionally, higher order genetic interactions between loci, leading to epistasis in complex traits may not be adequately captured by these traditional models (Taylor and Ehrenreich 2015; Varona et al. 2018). Machine learning (ML), a branch of computer science, addresses these limitations by capturing nonlinear interactions inherent in multi-omics data without relying on statistical assumptions (Greener et al. 2022).

ML identifies and extracts hidden biological features from high-dimensional datasets to predict traits for new, unseen data (Samuel 1959; Jordan and Mitchell 2015; Reel et al. 2021). It has been shown to provide accurate genotype-to-phenotype predictions for important agronomic traits by combining high-dimensional datasets such as environmental data, images, and genomic data (Danilevicz et al. 2022). ML has been successfully applied in plant disease resistance prediction using genomic information, identifying gene regulatory networks and predicting pathogen effector proteins (Sperschneider 2020; Upadhyaya et al. 2024). The integration of multi-omics data with advancements in artificial intelligence, including ML, into a crop genome design system termed “Breeding 4.0” has been proposed to enable data-driven decisions in plant breeding pipelines (Wallace et al. 2018; Jiang et al. 2020). However, despite its potential, the integration of multi-omics data with ML for disease resistance prediction in legumes remains largely limited. In legumes such as groundnut, breeding for disease resistance has been slow due to its large, tetraploid genome and limited applications of genomic tools (Zhang et al. 2017; Bangaru et al. 2023). Similarly, in faba bean, large genome size, low-density genetic maps, and insufficient development of tightly linked markers for disease resistance has made conventional breeding approaches less effective (Tiwari et al. 2023). Emerging approaches such as ML and multi-omics integration have the potential to accelerate resistance breeding in these and other underutilised legumes.

In this review, we discuss how integrating multi-omics with machine learning has the potential to improve disease resistance prediction in legumes. Multi-omics integration (MOI) in this review refers to combining more than one omics technology for analyses using machine learning models. We will briefly review various omics technologies, discuss machine learning approaches for multi-omics data integration and their applications in trait prediction.i

### Overview of multi-omics approaches

Plant-pathogen interactions involve a dynamic exchange in which plants defend against pathogenic attacks, while pathogens evolve mechanisms to disrupt plant defences. These interactions are mediated by molecules produced by both plants and pathogens, including proteins, sugars, small molecules (metabolites) and lipopolysaccharides, which can be captured using individual omics approaches (Boyd et al. 2013). Genomics characterises an organism’s entire genetic material, including both genes and non-coding regions, and provides insights into genetic diversity (Weissenbach 2016). It captures the genetic blue print of plants, identifying genes and genetic variants associated with resistance or susceptibility, such as resistance genes, QTLs, regulatory elements, and structural variations (Neik et al. 2020). Advances in NGS technologies and high-throughput phenotyping platforms (e.g. unmanned aerial vehicle-based imaging, hyperspectral sensors) have generated vast genomic and phenotypic datasets, enabling researchers to identify SNPs associated with traits and guide breeding strategies (Yang et al. 2020; Marsh et al. 2021). Plant-pathogen interactions are controlled by interconnected regulatory networks which can be captured using transcriptomics (Wani and Ashraf 2018). Transcriptomics quantifies RNA expression levels of transcripts in a cell, known as the transcriptome, including both coding messenger RNAs (mRNAs) and non-coding RNAs (Lowe et al. 2017). By using NGS technologies such as RNA sequencing (RNA-seq), transcriptomics captures gene expression changes across various tissues and cells under specific environmental conditions and developmental stages (Wilhelm et al. 2008; Wang et al. 2009). This approach is particularly useful in host–pathogen studies, as genome-wide transcriptional profiling of susceptible and resistant plants can reveal the molecular basis underlying plant defence responses at different stages of infection (Chen et al. 2013; Kamber et al. 2016; Kandel et al. 2020).

Epigenomics examines the complete set of heritable epigenetic processes such as DNA methylation, histone modifications, and alterations in chromatin structure that regulate gene expression without changing the underlying DNA sequence (Qureshi and Mehler 2011). These processes play an important role in plant defence responses by actively regulating defence-related gene expression during transcriptional reprogramming in response to pathogen attacks (Ding et al. 2012; Zhu et al. 2016; Zhi and Chang 2021). The correlation between mRNA and protein abundance varies greatly across organisms and cell types due to translational regulation (Plotkin 2010; Vogel and Marcotte 2012). This mRNA—protein correlation is often nonlinear and becomes even weaker under stress (Ponnala et al. 2014). Thus, relying solely on transcriptomics is insufficient for understanding protein functions in plant-pathogen dynamics. Proteomics provides an understanding of the expression, function and regulation of the proteins, or proteome, encoded by an organism, which helps to understand the post-translational modifications of proteins involved in plant defence responses (Zhu et al. 2003; Cembrowska-Lech et al. 2023). Metabolomics uses advanced spectroscopy techniques to identify metabolites in plants, and capture the flow of information from the genome through the transcriptome and proteome (Liu and Locasale 2017). Metabolic changes reveal interactions between small molecules and biochemical changes in response to stress, giving insights into metabolomic reprogramming during plant defence (Hong et al. 2016; Kumar et al. 2017). Comprehensive reviews covering potential applications of multi-omics approaches for crop improvement has been published previously (Yang et al. 2021; Zhang et al. 2022; Scossa et al. 2021).

## Machine learning approaches for trait prediction using multi-omics data

The availability of large and dense marker datasets in many plant species have not always translated to improvements in prediction accuracy in GS (Solberg et al. 2008; Norman et al. 2018), often requiring pre-selection of markers for improvement (Raymond et al. 2018). For instance, in sugarcane (*Saccharum sp.)*, machine learning models trained on the full genome-wide markers achieved only 50% prediction accuracy for brown rust resistance; however, reducing the marker set through refinement increased the accuracy up to 95% (Aono et al. 2020). Although GWAS have been instrumental in identifying loci associated with important plant agronomic traits, their use in phenotype prediction remains limited in some cases, as QTLs often explain only a small proportion of phenotypic variability, especially in polygenic traits (de los Campos et al. 2010; Hu et al. 2019). In wheat, ML models using only QTL-targeted markers showed lower prediction accuracies for Fusarium head blight-related traits, which improved after combining with genome-wide markers for broad genomic coverage (Rutkoski et al. 2012). These findings show that simply increasing marker density or refining marker set within a single-omics layer does not always yield optimal prediction performance, highlighting the need for more integrative approaches. Such differences in prediction performance could be attributed to trait heritability, linkage disequilibrium between markers, population structure, marker density, prediction models used, and genetic architecture of the trait where the loci influencing the trait may not show purely additive effects (Kaler et al. 2022; Zhang et al. 2019). Additionally, genomic sequence information alone may not be able to capture the impact of downstream regulatory processes and gene interactions on phenotype (Zhu et al. 2012; Ritchie et al. 2015). Other single-omics layers such as transcriptome and metabolome have been explored in predictive models, with their effectiveness varying based on trait and species. Metabolic-based predictions showed low accuracy than SNPs for yield prediction in barley (*Hordeum vulgare* L.) (Gemmer et al. 2020); however, transcriptome-based models have achieved up to 92.86% accuracy in classifying disease stress-responsive genes in maize (*Zea mays* L.) (Nazari et al. 2023). In some cases integrating biological information from omics layers such as transcriptomics and metabolomics along with genomic features into a single model has been shown to improve prediction accuracy using statistical and ML models, in various plant species (Table 1). However, the relative performance of multi-omics data in prediction models may depend on the specific trait and species under study. A combination of genomic and metabolomic data showed higher prediction power in rice (Wang et al. 2019), while combining mRNA and genomic data improved prediction in maize (Schrag et al. 2018).

**Table 1:** Recent studies integrating multi-omics data for trait prediction in plants

Crop	Trait	Multi-omics data	Model	Performance	References
Rice (*Oryza sativa* L.)	Yield	G, T, M	MLASSO	G + T + M improved predictive ability to 0.2451 from 0.1588 with G alone	(Hu et al. 2019)
Rice	Yield,1000-grain weight, grains/panicle, tillers/plant	G, M, T	BLUP and other seven statistical models	G + M achieved the best prediction for all four traits. BLUP was the most efficient prediction method	(Wang et al. 2019)
Oat (*Avena sativa* L.)	Agronomic traits (17 traits)	G, T, M	GBLUP, multi-trait models	G + T + M improved prediction accuracy for all traits in single environment. Multi-trait omics models outperformed multi-trait GBLUP in multi-environment	(Hu et al. 2021)
Barley (*Hordeum vulgare* L.)	Leaf angle, Heading time, Plant height	G, T, M	GBLUP	T + M improved prediction ability over single predictors, with trait-specific optimal weights	(Wu et al. 2022)
Arabidopsis (*Arabidopsis thaliana* L.)	Complex traits including flowering time	G, T, Me	rrBLUP, RF	G + T and G + T + Me improved RF model performance and revealed additional gene interactions	(Wang et al. 2024a)
Maize (*Zea mays* L.)	Yield	G, M, Ph	Linear regression-based and ML models	G + M + Ph improved prediction accuracy from 0.32 to 0.43 in RF	(Wu et al. 2024)

Abbreviations: G, Genomic data; T, Transcriptomic data; M, Metabolomic data; Me, Methylomic data; E, Epigenomic data; P, Proteomic data; Ph, image-based phenomic data; MLASSO, Multilayered Least Absolute Shrinkage and Selection Operator; BLUP, Best Linear Unbiased Prediction; GBLUP, Genomic Best Linear Unbiased Prediction; rrBLUP, ridge regression Genomic Best Linear Unbiased Prediction; RF, Random Forest; AUC-ROC, Area Under the Curve of the Receiver Operating Characteristic

The different ML approaches that can be used for MOI are supervised and unsupervised learning (Reel et al. 2021). The goal of supervised learning is to build predictive models for new data using labelled data with known genotype–phenotype relationships (e.g. resistant vs. susceptible) (Danilevicz et al. 2022). Commonly used supervised learning algorithms include Support Vector Machines (SVM) (Cortes and Vapnik 1995), Random Forest (RF) (Breiman 2001), Gradient tree boosting (Friedman 2001) and neural networks (McCulloch and Pitts 1943). SVM can identify nonlinear patterns present in multi-omics data through the use of kernel functions, and they are particularly effective when the number of features such as genes or proteins exceeds the number of samples. Ensemble learning methods such as RF generate multiple decision trees based on random subsets of features and averages their prediction to improve accuracy (Breiman 2001). RF ranks the importance of each feature within a model, making it useful for identifying genes or metabolites contributing to the prediction outcomes. RF captures nonlinear relationships between genotypes and traits, and is resistant to overfitting, making it well suited for high-dimensional multi-omics data analysis (Mukherjee et al. 2024). Unsupervised learning methods identify hidden patterns and structures within multi-omics input data using unlabelled data, without relying on pre-defined trait labels. This approach is helpful to reveal novel associations or groupings related to disease resistance that may not be apparent through traditional analysis methods (Shomorony et al. 2020). Unsupervised methods based on regression, clustering, or network-based have been developed for multi-omics data integration (Vahabi and Michailidis 2022).

Deep learning (DL), a subset of ML, is based on the information processing patterns present in the human brain and uses neural networks to learn from data (Alzubaidi et al. 2021). DL models, especially deep neural networks have been used for MOI due to their ability to identify nonlinear patterns and reduce dimensionality (Krassowski et al. 2020; Kang et al. 2022). Among DL methods, graph neural networks (GNNs) are well suited for MOI as GNNs use a graph architecture for representing data, with nodes representing molecules (e.g. genes, proteins), and edges representing interactions between these molecules (Valous et al. 2024). This architecture allows different omics data to be integrated into a single graph structure, facilitating comprehensive analysis of biological systems (Valous et al. 2024). Gene network prediction model (NetGP) is a DL approach that integrates transcriptomic, genomic and multi-omics data for plant phenotypic prediction (Zhao et al. 2025).

Accessible web-based deep learning tools, such as G2PDeep-v2, enable phenotype prediction in plants using multi-omics data, including copy number variation, gene expression and genetic markers, through algorithms including SVM, RF and multi-convolutional neural networks (Zeng et al. 2024). Multimodal deep learning approaches can be used to integrate additional information such as environmental data or infection-stage images into multi-omics data. Multimodal deep learning enables the fusion of data from different sources and types including images, text and high-dimensional data into a single model (Gao et al. 2020). This approach has the potential to integrate multi-omics data with environmental factors to provide robust prediction accuracy for implementing GS in breeding programmes (Montesinos‑López et al. 2024).

### Characteristics of multi-omics data and integration strategies

**Fig. 1:**
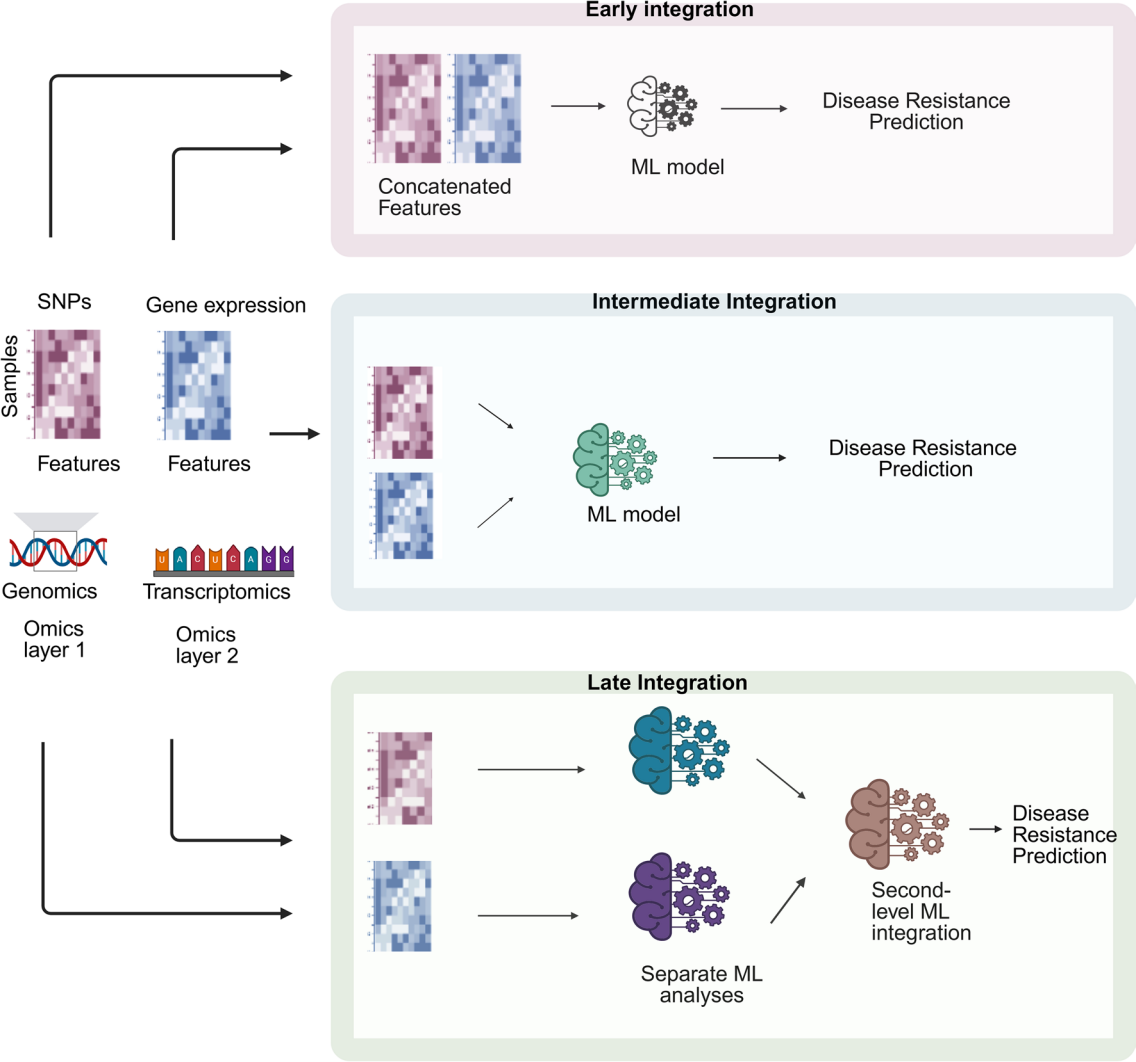
Illustration of different integration strategies for multi-omics data. In early integration, features from different omics are concatenated into a single matrix before being fed into the ML model. In intermediate integration, ML algorithms learn a joint representation of the dataset during the analysis stage to develop predictions, preserving the unique characteristics of each dataset. In late integration, outputs from the separate analysis of each omics dataset are used to train a second-level model, which is used to make the final prediction (Zitnik et al. 2019). Figure created with BioRender.com (Ikbal 2025). Abbreviations: ML, machine learning; SNPs, single-nucleotide polymorphisms

Biological data from different omics layers typically show high dimensionality, where the number of features is much larger than the number of samples (large *p,* small *n*) (Feldner-Busztin et al. 2023). This high dimensionality can lead to overfitting or spurious associations between genotypes and phenotypes, reducing the generalisability of the data (Athieniti and Spyrou 2023). Omics data vary in their data types (e.g. continuous, numerical, categorical) and statistical distributions, often with high noise levels, leading to data heterogeneity (Athieniti and Spyrou 2023). Biological data also contains highly correlated features due to strong underlying relationships, leading to redundancy when overlapping information is captured across layers (Li et al. 2018). Additionally, data for complex traits such as disease resistance suffer from class imbalance with uneven distribution of phenotypic classes (e.g. unequal representation of resistant vs. susceptible individuals) (Picard et al. 2021). Moreover, certain omics data, such as gene expression, protein abundance, or metabolite levels are inherently non-negative, which needs to be preserved throughout data analysis. These challenges require advanced ML models and specialised MOI strategies to ensure robust analyses (Fig. 1).

Depending on when the multi-omics data are integrated into a machine learning process, MOI strategies can be classified as early, intermediate (joint), or late integration (Fig. 1) (Zitnik et al. 2019). Early integration combines features, which are individual measurable properties associated with a phenotype, from different omics datasets into a single vector before feeding it into a ML model (Zitnik et al. 2019). This approach treats all features as independent without capturing the relationships between the features from different omics layers (Zitnik et al. 2019). In intermediate integration, the algorithm learns a common representation of the data during the analysis stage while maintaining the unique characteristics of each dataset (Zitnik et al. 2019). This approach does not combine raw input data as in early integration or develop separate models for each dataset but uses algorithms such as multiple kernel learning or deep learning to form a unified representation of the data (Zitnik et al. 2019). Intermediate integration often gives superior performance and allows the model to leverage the relationships across omics datasets, making it particularly effective for predicting complex traits such as disease resistance (Zitnik et al. 2019). The most straightforward strategy is late integration, in which models are initially built separately for each omics data, and then combined by training a second-level model (Zitnik et al. 2019). However, late integration fails to capture interactions among different omics layers (Picard et al. 2021). Comprehensive reviews of MOI for machine learning in various systems have been published previously (Subramanian et al. 2020; Reel et al. 2021; Picard et al. 2021).

### Feature selection and dimensionality reduction

Regardless of the integration strategy, multi-omics datasets require dimensionality reduction to manage the large number of variables, reduce noise, and improve the accuracy of analyses (Meng et al. 2016). For instance, in high-dimensional gene expression data, a number of features or genes can be highly correlated (Clarke et al. 2008). Similarly, significant SNPs identified through GWAS models that do not consider linkage disequilibrium between SNPs may often be linked, causing redundancy (Pudjihartono et al. 2022). As correlated features explain the same information about the data, retaining one of the features is sufficient for phenotype prediction (Chandrashekar and Sahin 2014). In multi-omics datasets, correlated features across omics layers may add more complexity. The extra variables describing redundant information, serve as noise in the dataset, often reducing predictor performance (Kubus 2019). Feature selection (variable elimination) is a dimensionality reduction technique that addresses this issue. It involves selecting a subset of relevant features that explain most of the variance in the dataset, thereby reducing redundant variables (Guyon and Elisseeff 2003). This subset is then used to train the model. Feature selection reduces the risk of overfitting, as models with fewer, more relevant features are less likely to fit noise into the training data, leading to improved prediction on unseen data (Guyon and Elisseeff 2003).

For multi-omics data, selected features might include genomic data such as SNPs associated with disease resistance, and gene expression profiles to identify genes activated during pathogen attack (Liu et al. 2024; Michel et al. 2021). Epigenetic features such as histone modifications, global DNA methylation levels, and genome-wide chromatin accessibility can provide insights into the epigenomic regulatory landscape during plant-pathogen interactions (Ramirez‑Prado et al. 2018). Proteome features, including protein abundance levels, post-translational modifications, and metabolomic features such as labelled plant metabolite levels involved in defence pathways can further contribute to building a comprehensive multi-omics model for predicting disease resistance (Castro‑Moretti et al. 2020; Elmore et al. 2021). However, in the case of disease resistance prediction in legumes, selecting features based only on their known associations with resistance through supervised feature selection may introduce bias (Ambroise and McLachlan 2002). This may lead to overfitting, as the ML model might only learn from these specific features resulting in reduced performance for new unseen data (Ambroise and McLachlan 2002). Approaches such as using a variance filter through unsupervised feature selection to select features that are highly variable across all the samples without relying on their disease resistance status have been used previously to reduce bias and improve generalisability (Bommert et al. 2022).

Before training the model, the selected features from various omics layers must be pre-processed and normalised to prevent biases that could arise due to difference in data scale or distributions (Picard et al. 2021). There are numerous feature selection methods available varying in their underlying principles (Effrosynidis and Arampatzis 2021). These methods are classified into filter, wrapper, and embedded methods depending on how the feature selection process interacts with the learning algorithm (Effrosynidis and Arampatzis 2021). The method to be chosen for multi-omics datasets depends on several factors including performance of the method, computation time, dataset characteristics and size (Li et al. 2022). Hybrid methods integrating multiple feature selection techniques through ensemble approaches may give better performance for multi-omics datasets (Claude et al. 2024).

## Machine learning applications in multi-omics integration for predicting disease resistance

### Integrating single-nucleotide polymorphisms, and genomic features for machine learning-based trait prediction

Advances in genomic technologies and research have enabled characterisation of genetic diversity and resistance loci associated with numerous diseases in a number of legume species (Gangurde et al. 2022). Trait-associated regions identified through GWAS can be refined using local haplotyping by detecting haplotype structures and the linkage landscape surrounding the causal variants within specific genomic regions (Marsh et al. 2023). The stacking of beneficial haplotypes associated with multiple diseases has been shown, through simulation studies, to improve disease resistance (Tong et al. 2024). The development of pangenomes has further enhanced disease resistance studies. Pangenomes reduce reference bias to improve trait prediction accuracy (Danilevicz et al. 2020). They capture the genetic diversity within a species beyond a single reference genome, by identifying both the core conserved genome present in all individuals, and the dispensable genome absent in some individuals (Danilevicz et al. 2020; Bayer et al. 2020). This is important for crop improvement, as rare beneficial alleles present in a few individuals or wild crop relatives can be introduced to domesticated varieties that lack these alleles (Tay Fernandez et al. 2022). Pangenomes can facilitate the transfer of knowledge from major crops to underutilised crops (Hu et al. 2025) and uncover novel resistance alleles absent from reference genomes, providing breeders with new genetic diversity to improve disease resistance (Golicz et al. 2016; Bayer et al. 2019). The construction of a chickpea super pangenome enabled the identification of a catalogue of R genes, providing a valuable resource for breeding disease-resistant varieties (Khan et al. 2024). Insights from genomics have facilitated the breeding of resistant legumes, such as fusarium wilt-resistant chickpea (Mannur et al. 2019), improved late leaf spot and rust resistant groundnut (*Arachis hypogaea* L.) (Ramakrishnan et al. 2020), and generated elite common bean (*Phaseolus vulgaris* L.) varieties with multiple disease resistance and high seed iron (Portilla et al. 2022), and forms a foundation for ML-based predictive modelling.

The growing availability of genomic resources have enabled their use in ML models for plant disease resistance prediction using features such as pre-selected SNPs, haplotypes, image-derived traits and environmental covariates (Upadhyaya et al. 2024). For bacterial wilt resistance in common bean, whole genome-wide SNPs (*n* = 4568) and preselected SNPs (*n* = 14) were used for genomic prediction with seven models, including RF and SVM (Zia et al. 2022). Prediction accuracy ranged from 0.37 to 0.58 with the full SNP set and 0.30–0.53 with the preselected SNPs in different models. However, only for one bacterial wilt isolate, the accuracy was higher with the selected SNP set in RF model (Zia et al. 2022). Haplotype blocks have the potential to improve prediction accuracy by capturing local epistasis between markers, though the extent of improvement is often trait-, species-, and model-dependent (Weber et al. 2023; Lin et al. 2024). In wheat, LD-based haplotype-tagged SNPs improved prediction accuracy for Fusarium head blight resistance showing correlation higher than models using non-pre-selected SNPs (Alemu et al. 2023). Moreover, genotype-environment interactions that impact phenotypic variability can be effectively captured in ML models through feature engineering that combines genomic and environmental data, thereby enhancing prediction accuracy as shown in maize and adaptable for disease resistance studies in legumes (Fernandes et al. 2024). Beyond genomic information, transcriptomic data provides dynamic changes in gene expression in response to a disease and has the potential to improve predictive power (Michel et al. 2021).

### Transcriptomic and genomic feature integration for disease resistance prediction

RNA-seq studies have identified differentially expressed genes and pathways involved in various legume-pathogen interactions. For instance, comparative transcriptome profiling in susceptible and resistant chickpea infected with *Fusarium oxysporum* f.sp. *ciceri* (Foc) revealed that Foc genes necessary for pathogen survival are present only in susceptible cultivars, providing potential targets for pathogen intervention and disease management strategies (Upasani et al. 2017). Similarly in common bean, transcriptome profiling identified upregulated sugar transporter genes in susceptible lines during early stages of *Colletotrichum lindemuthianum* (Sacc. & Magnus)-Briosi & Cavara. infection (Padder et al. 2016). Moreover, integrating publicly available data from GWAS and transcriptomics have led to identifying candidate genes associated with immunity-related cellular processes against five fungal pathogens in soybean, underscoring the potential of multi-omics data integration to provide insights into disease resistance mechanisms (Almeida‑Silva and Venancio 2021).

ML methods have the potential to improve the accuracy of transcriptomic analyses by identifying novel defence-related gene expression patterns and regulatory interactions, compared to conventional methods (Panahi et al. 2025). For instance, RNA-seq, in vitro transcription factor-DNA profiling and DL were integrated to study the soybean defence response to *Phytophthora sojae* Kaufmann and Gerdemann infections (Hale et al. 2023). Two defence-related transcription factors (TFs) (WRKY and RAV families) were selected from the differentially expressed genes and their binding sites were identified using DAP-seq. These binding sites were used to train Convolutional Recurrent Neural Networks (CRNNs), which predicted novel TF binding sites across the soybean genome with 89–90% test accuracy (Hale et al. 2023). The study further leveraged the homology between Arabidopsis and soybean to build CRNNs for other defence-related TF families with high performance accuracy (> 90% accuracy) in cross-family predictions and moderate accuracy of 60% for Arabidopsis-to-soybean predictions (Hale et al. 2023). Another application of ML on transcriptomic data was demonstrated by Sia et al. (2025) who used transcriptional patterns derived from early stages of infection to predict final disease outcomes across pathosystems. Using a dual-species RNA-seq data for Arabidopsis-*Botrytis cinera*, Sia et al. (2025) developed multiclass classification of disease severity using SVM, RF, XGBoost and Deep Neural Network which were trained on a subset of the transcriptome using 12 feature selection techniques including domain knowledge and co-expression network measures. XGBoost achieved the highest accuracy (72.3%), when trained on dual-species transcripme data to predict final disease outcome (Sia et al. 2025). They further show that pretrained models on reduced feature selection gene sets from Arabidopsis-*B.cinera* interaction can predict disease outcomes for Arabidopsis-*Sclerotinia sclerotiorum* with RF showing the highest 1-class error accuracy (Sia et al. 2025). The study further demonstrated transfer learning, applying the pretrained models with the fungal interaction to predict Arabidopsis-*Pseudomonas syringae* bacterial infections.

Integrating genomic data with RNA-seq has been shown to further improve prediction accuracy. A machine learning algorithm, Integrated Multi-Omics Analysis and Machine Learning for Target Gene Prediction (iMAP), was developed to predict calcium signalling genes within resistance-associated loci influencing *Sclerotinia sclerotiorum* resistance in oilseed rape (*Brassica napus* L.) (Wang et al. 2024b). The iMAP framework is a prime example of how multi-omics data including genomic, transcriptomic, and functional annotations can be pre-processed, integrated, and used in ML algorithms to improve resistance gene prediction compared to single-omics approaches. The authors first pre-processed genomic data using PLINK v1.9 (Purcell et al. 2007), and conducted GWAS using three statistical models in rMVP v1.0.0 R-package (Yin et al. 2021). This was followed by haplotype-based GWAS in RAINBOWR v0.1.36 R-package (Hamazaki and Iwata 2020) and weighted gene co-expression network analysis (WGCNA) using RNA-seq data. They also compiled gene function annotations for the top significant SNPs and differentially expressed genes from public databases (Wang et al. 2024b) Features were extracted from each data layer including single-SNP GWAS statistics, haplotype GWAS results, WGCNA, gene ontology functional annotations, and combined to a high-dimensional matrix using an early integration strategy. Principal component analysis was used for dimensionality reduction, retaining 99.5% variance. ML models including RF, SVM, XGBoost and logistic regression were trained on these features using scikit-learn library in Python (Wang et al. 2024b). When using only single-SNP GWAS features, RF model showed an accuracy of 0.78 and F1 score of 0.69. However, the RF model using the combined features consistently outperformed single-omics features, with a highest accuracy of 0.95 (Wang et al. 2024b). The iMAP algorithm using the RF model and the combined features also predicted seven calcium signalling genes within three resistance-associated loci on chromosome A06 (Wang et al. 2024b), showing the potential of MOI for improving the prediction of quantitative disease resistance mechanisms and suggests that similar strategies could be effective for legumes.

### Epigenomic data for trait prediction using machine learning

Advancements in third generation NGS technologies, such as nanopore and PacBio DNA sequencing, have enabled genome-wide epigenetic profiling by detecting DNA base modifications without the need for chemical treatments that could degrade DNA (Liu et al. 2019; Tse et al. 2021). As part of their defence strategy, plants can actively demethylate promoters and regions around defence-related genes, leading to increased gene expression to enhance protection (Annacondia et al. 2021). Such differentially methylated regions have been identified in *Arabidopsis thaliana (L.) Heynh* in response to *Pseudomonas syringae* pv. *tomato* DC3000 infection using whole-genome bisulfite sequencing (Dowen et al. 2012). Genome-wide epigenomic and transcriptomic changes in common bean-rust *(Uromyces appendiculatus)* interactions revealed differential acetylation and methylation patterns modulating gene expression in defence-related genes (Ayyappan et al. 2015). Similarly, in soybean, a combined transcriptomic and epigenomic study identified differentially expressed genes associated with defence pathways in resistant lines following infection by *Phytophthora sansomeana*. The study also found that increased CHH methylation levels (where cytosine is followed by non-guanine bases (A,T, or C)) in long intergenic non-coding RNAs suggest that specific methylation patterns may play a role in soybean’s defence against *Phytophthora sansomeana* (Lee et al. 2024). Thus, understanding epigenomic variations, such as DNA methylation patterns, in plant-pathogen interaction provides insights into disease resistance mechanisms in crops (Tirnaz and Batley 2019).

However, despite this growing understanding of the role of epigenetic mechanisms in plant defence response, ML studies integrating genome-wide epigenomic data for disease resistance prediction remains largely limited. For other complex traits including flowering time, integrating genomic, transcriptomic, and methylomic data has improved prediction accuracy using RF model revealing additional gene interactions (Wang et al. 2024a, Table 1). Similarly, in barley, including genomic, DNA methylation, and gene expression data in ML model enhanced complex trait prediction including grain yield and nitrogen uptake, explaining a greater proportion of phenotypic variance (0.72–0.92) than genomic models alone (0.55–0.86) (Hansen et al. 2022). Potential of epigenomic data for plant trait prediction has been shown in balsam poplar trees (*Populus balsamifera* L.) where artificial neural networks explained 57.5% and 40.9% phenotypic variance for biomass and wood density respectively (Champigny et al. 2020). Moreover, several ML and DL frameworks for predicting DNA methylation sites have been developed such as Deep6mAPred (Tang et al. 2022), MuLan-Methyl (Zeng et al. 2022), Methyl-GP (Xie et al. 2025) and AMPS (Sereshki et al. 2023) that uses genomic annotations to improve methylation prediction accuracy. Recently, a cross-species hybrid ML model showed that gene expression could be predicted from DNA methylation patterns in fungi, with the authors proposing its adaptation across species including plants (Weinstock et al. 2024). Future efforts are needed to understand the impact of epigenomic data in ML pipelines for predicting novel insights in legume-pathogen interactions.

### Proteomics for predictive trait modelling

Proteomics often complements transcriptomics, using high-throughput technologies such as mass spectrometry, providing insights into molecular mechanisms associated with plant stress response (Lan et al. 2012). For example, a combined transcriptomic and proteomic approach showed that yellow mosaic virus infection in soybean leads to differential expression of genes associated with cell wall biosynthesis, photosynthesis, stress and metabolism pathways (Pavan Kumar et al. 2017). A similar combined approach revealed that inoculating soybean with arbuscular mycorrhizal fungi promotes symbiosis, increasing resistance to root rot. This approach provided insights into the molecular mechanisms by which arbuscular mycorrhizal fungi promotes resistance through the upregulation of proteins such as phenylalanine ammonia-lyase, calcium-dependent protein kinase, and other defence-related proteins (Zhang et al. 2020b). Proteomics provides insights into molecular features that can be targeted for crop improvement, by aiding in the identification of protein biomarkers and cellular mechanisms involved in plant stress responses (Mustafa and Komatsu 2021).

Proteomic, genomic, transcriptomic, and metabolomic features were extracted and used for prediction of genes encoding enzymes in plant specialised metabolite synthesis in Arabidopsis using an automated ML framework, AutoGluonTabular (Bai et al. 2024) (Table 2). In this study, models trained only on proteomic and genomic features outperformed (AUC-ROC > 0.881) models that were trained using all the multi-omics features. Bai et al. (2024) also evaluated cross-species prediction by training models on Arabidopsis, maize, and tomato (*Solanum lycopersicum* L.) using genomic and proteomic features. A three-species model outperformed single-species prediction with an accuracy of 0.844, showing the value of shared molecular features across species (Bai et al. 2024). A graph-based DL technique, Weighted Gene Autoencoder Multi-Omics Relationship Prediction was used to analyse and predict regulatory interactions between the fungal pathogen, *Magnaporthe oryzae Oryzae* and rice, by integrating genomic, transcriptomic and proteomic data (Zhao et al. 2023). Despite the growing availability of proteomic data, their use in ML frameworks to uncover novel post-translational mechanisms associated with disease resistance remains largely unexplored in plants, unlike human disease studies (Geddes-McAlister and Uhrig 2025). A study in human medicine has shown that deep learning can classify disease states directly from proteomic data obtained through data-independent acquisition mass spectrometry, without peptide or protein identification (Zhang et al. 2020a; Meyer 2021). This approach of converting data matrices to image-like tensors and using convolutional neural networks for classification of healthy and diseased states achieved superior performance compared to models based on identified proteins (Zhang et al. 2020a) and similar strategies can be adapted in legume systems for disease classification.

**Table 2:** Studies integrating multi-omics data using machine learning for trait prediction in plants

Crop/ pathosystem	Omics layer	ML method	Trait predicted	Accuracy/metrics	References
Common bean/ *Curtobacterium flaccumfaciens* pv. flaccumfaciens (Cff)	G	Seven models including RF, SVM	Bacterial wilt resistance	Up to 0.58 (full SNPs), up to 0.53 (preselected SNPs)	(Zia et al. 2022)
Soybean/ *Phytophthora sojae*	T, DAP-seq	CRNN (DL)	TF binding site prediction	89–90% test accuracy	(Hale et al. 2023)
Arabidopsis/*Botrytis cinera,* Arabidopsis/*Sclerotinia sclerotiorum* & *Pseudomonas syringae*	Dual RNA-seq	SVM,RF, XGBoost, DNN	Disease class prediction based on early transcriptional response	All models outperformed whole transcriptome-only baseline	(Sia et al. 2025)
Canola/*Sclerotinia sclerotiorum*	G,T, functional annotation	RF, SVM, XGBoost,LR	Resistance to *S.sclerotiorum*	RF with combined features: accuracy 0.95; SNP-only: 0.78	(Wang et al. 2024b)
Barley	G,T,E	BGAM	Complex traits	G + T + E 0.72–0.92 PVE, G only -0.55–0.86 PVE	(Hansen et al. 2022)
Multispecies	G,T,E,P	AutoGluon-Tabular	Metabolite biosynthesis	P and G features achieved the highest accuracy (AUC-ROC > 0.881	(Bai et al. 2024)
Rice/ *Magnaporthe oryzae Oryzae*	G,T,P	WGCNA and graph autoencoder	Relationship prediction for rice and pathogen	Predicted for multi-omics relationship data	(Zhao et al. 2023)
Citrus/*Candidatus Liberibacter asiaticus*	M, microbiome sequencing	RF, Gradient Boost, Adaboost	Differentially expressed metabolites & bacteria	RF accuracy up to 97.22% ± 2.27%	(Li et al. 2023)

Abbreviations: AUC-ROC, area under the curve of the receiver operating characteristic; BGAM, Bayesian generalised additive models; CRNN, convoluted recurrent neural network; DAP-seq, DNA affinity purification sequencing; DL, deep learning; E, epigenomic data; G, genomic data; LR, logistic regression; M, metabolomic data; ML, machine learning; P, proteomic data; PVE, phenotypic variance explained; RF, random forest; RNA-seq, RNA sequencing; SNP, single-nucleotide polymorphisms; SVM, support vector machines; T, transcriptomic data; TF, transcription factor; WGCNA, weighted gene co-expression network analysis

### Integrating metabolomics with other omics layers for trait prediction

Metabolites are involved in enzyme regulation and can enhance the plant’s ability to respond to pathogen attack. For example, metabolomic analysis identified specific sugars and secondary metabolites in soybean resistant to *Phytophthora sojae* Kauffmann and Gerdemann, indicating their potential roles in defence (Zhu et al. 2018). Metabolomics often complements data from other omics layers to reveal pathogen infection mechanisms (Peyraud et al. 2017; Castro‑Moretti et al. 2020). A combined transcriptomic and metabolomic analysis showed that *Fusarium solani f. sp. phaseoli* infection in common bean leads to cell wall modification and reactive oxygen species generation, highlighting their roles in defence (Chen et al. 2020). Metabolomic approaches have also identified biomarkers including palatinitol and L-proline, which contribute to *Fusarium oxysporum f. sp. lentis* resistance in lentils (Foti et al. 2024). Large-scale metabolomic data can be integrated with genomic information to perform metabolite GWAS, enabling the identification of candidate genes associated with specific metabolite profiles (Fang and Luo 2019). In legumes, a metabolic and lipidomics atlas has been developed for multiple species, including chickpea and lentil that can facilitate the study of metabolites involved in disease resistance through metabolite-based GWAS (Bulut et al. 2023).

Metabolomics, when integrated with other multi-omics data has the potential to enhance the understanding of gene regulatory networks, protein interactions and pathways involved to link genotypes to phenotypes (Hao et al. 2025). In a study on huanglongbing-affected citrus cultivars, eight supervised ML classifiers including RF, Gradient Boost and Adaboost, were used for feature selection on metabolomics and microbiomics sequencing data to identify differentially expressed metabolites and bacteria (Li et al. 2023). RF showed a testing accuracy up to 97.22% ± 2.27% in this study. The ML results were further integrated with the Kyoto Encyclopedia of Genes and Genomes pathway enrichment methods and correlation analysis to identify potential interactions between plant metabolic pathways, such as ABC transporters, which play a major role in plant defence (Li et al. 2023). Similarly, for complex traits such as grain yield, integrating metabolomic, genomic data and phenotyping images from different developmental stages, increased the prediction accuracy in maize from 0.32 to 0.43 using an RF model (Wu et al. 2024). When evaluating feature importance, RF models ranked the metabolite markers higher than the genomic markers, suggesting that continuous quantitative traits provided by metabolites contain more information for predicting yield than binary markers (Wu et al. 2024). These findings show that RF is particularly effective at handling complex interactions within multi-omics data with higher prediction accuracy (Wu et al. 2024).

### Integration of phenotypic imaging with multi-omics data for disease resistance prediction

Recent advancement in phenotyping include automated systems, high-throughput methodologies for large-scale testing, and fine-scale phenotyping of specific organs or tissues at high resolution (Zhao et al. 2019). These high-throughput and automated approaches enable efficient, non-destructive assessment of plant stress response, capturing the dynamic expression of traits and generating temporal and spatial data, including images (Gill et al. 2022). Imaging-based phenotyping has improved disease evaluations, and can be integrated into ML and DL frameworks, to enable models to learn features distinguishing healthy from diseased phenotypes (Dolatabadian et al. 2025). For example, visible and thermal imaging were used to train ML models to predict wilt severity in chickpea under field conditions (Sing et al. 2023b). Visible RBG imaging detects visible disease symptoms such as leaf discolouration, lesions, and wilting, while thermal imaging captures changes in surface temperature, stomatal closure and water stress responses (Zhao et al. 2019). In the study by Singh et al. (2023b), twelve ML models including SVM, RF, Cubist, and k-nearest neighbours were trained on image-derived features, with model combination techniques which further improved wilt severity prediction. Additionally, Bai et al. (2023) used time-series remote sensing data from unmanned aerial vehicles along with accumulated temperature data to train ML models for bacterial blight prediction model in rice. Support vector regression using both spectral and auxiliary traits showed the highest predictive accuracy (Rp^2^ = 0.85) across geographical locations (Bai et al. 2023). Integration of multi-omics data with image-derived traits has also been applied to predict complex traits such as yield (Table 1). For instance, Wu et al. (2024) combined genomic, metabolomic, and high-throughput imaging data with ML approaches for maize yield prediction. An explainable deep learning framework (EG-CNN) that integrates multi-omics data with hyperspectral imaging has also been developed for plant disease classification (Shoaib et al. 2023). The model trained on gene expression profiles, metabolite abundances, and hyperspectral images for four diseases (powdery mildew, rust, leaf spot, blight), achieving 95.5% accuracy in classifying disease types (Shoaib et al. 2023). Notably, the results of the EG-CNN model were explained using saliency maps, which shows the regions of the input data that contribute most to the model’s output and decision-making. Extending DL models such EG-CNN to legumes could further enhance our understanding of underlying biological mechanisms in diseases.

These studies collectively show the potential of multi-omics and machine learning approaches to understand the complex biological mechanisms behind legume-pathogen interactions for resistance improvement, as well as public resources available for such analyses (Table 3). However, not all machine learning models, integration strategies, or omics data type perform equally well across different datasets or prediction tasks (Picard et al. 2021). Adding more omics layers may introduce noise and redundancy, and optimal integration requires combining biological knowledge along with machine learning approaches (Picard et al. 2021). Prediction accuracy can vary widely depending on data type, integration strategies, specific biological questions, necessitating careful benchmarking and model selection for multi-omics data integration (Flores et al. 2023; Picard et al. 2021).

**Table 3:** Publicly available tools and pipelines for multi-omics integration and trait prediction

Name	Description	Focus	Github link
awesome-multi-omics	Curated list of multi-omics integration software, including many ML-based prediction methods	General	https://github.com/mikelove/awesome-multi-omics
IntegratedLearner	R-package for multi-omics prediction and classification	General	https://github.com/himelmallick/IntegratedLearner (Mallick et al. 2024)
DEM	A multimodal deep learning architecture for phenotypic prediction and functional gene mining of complex traits	Plant	https://github.com/cma2015/DEM/ (Ren et al. 2024)
DNNGP	Deep neural network-based method for genomic prediction using multi-omics data in plants	Plant	https://github.com/AIBreeding/DNNGP (Wang et al. 2023)
NetGP	Gene network-based multi-omics integration for genomic prediction in plants	Plant	https://github.com/pingT-researcher/NetGP (Zhao et al. 2025)
panomiX	Platform for multi-omics feature analysis and prediction in plants	Plant	https://github.com/NAMlab/panomiX-tool (Sahu et al. 2025)
Prediction of plant complex traits via integration of multi-omics data	A study integrating genomic, transcriptomic, and methylomic data for complex trait prediction	Plant	https://github.com/ShiuLab/2024_Ath_GP (Wang et al. 2024a)
AttentionMOI	Deep learning algorithm for multi-omics integration to predict cancer subtypes and survival	General	https://github.com/BioAI-kits/AttentionMOI
Integration of multi-omics data and deep phenotyping provides insights into responses to single and combined abiotic stress in potato	A study on integrative multi-omics data analysis using a bioinformatics pipeline based on ML and knowledge networks	Plant	https://github.com/NIB-SI/multiOmics-integration (Zagorščak et al. 2025)
MultiOmicsIntegrator	A Nextflow pipeline for integrated omics analyses	General	https://github.com/Bianca-Pasat/MOI (Pasat et al. 2024)
MOVE	Multi-omics variational autoencoder frame for multi-omics integration and identification of cross modal associations	General	https://github.com/RasmussenLab/MOVE (Allesøe et al. 2023)

## Challenges in using machine learning with high-dimensional multi-omics data and future perspectives

Multi-omics data pose several challenges in ML models, due to differences in normalisation, scaling, and data distribution across different layers. For example, metabolomics data may be assigned null values when measurements fall below detection levels (Antonelli et al. 2019). This can be overcome through imputation for missing data including for genotype, epigenomic and proteomic datasets (Song et al. 2020). In disease resistance studies, class imbalance in phenotype values can lead to overfitting in ML models trained using such imbalanced datasets. This can be overcome by over-sampling the minority class or under-sampling the majority class, generating synthetic data using methods such as the synthetic minority oversampling technique, adaptive synthetic sampling, or by measuring the performance of the model based on weighted or normalised metrices instead of relying solely on the accuracy of the model (He et al. 2008; Jeni et al. 2013; Blagus and Lusa 2013; Reel et al. 2021).

Additionally, ML and DL models require a large volume of data to learn from as their predictive performance decreases with smaller datasets, and this data volume may not be available for all omics layers (Dou et al. 2023). Strategies such as transfer learning, where a model is trained on a source task with abundant data to learn features, and then applied to a target task with less data for prediction can be employed in such cases (Pan and Yang 2010; Weiss et al. 2016). For example, TrG2P, a transfer learning framework has been used for yield prediction by pre-training the model with non-yield traits using convolutional neural networks, and validated in rice, maize, and wheat (Li et al. 2024). The success of ML for MOI also depends on access to high-quality, well-annotated omics datasets that can include features such as histone modifications, chromosomal interactions, and metabolic pathways. Databases such as SoyMD and SoyOmics provide omics datasets for studies in soybean, but expanding such resources to underutilised legumes will help to advance MOI for disease resistance prediction (Liu et al. 2023; Yang et al. 2024). Deploying ML and DL models requires high-performance computing, cloud storage, and efficient data-sharing platforms (Luo et al. 2024). This poses challenges to sustainable deployment and necessitates collaboration and resource sharing among the various sectors for efficient plant disease management (Jeger et al. 2024).

While extensive multi-omics databases are being developed, legumes, particularly underutilised crops such as pigeon pea and cowpea, lack the comprehensive resources available for other major crops. Mining relevant information from existing databases to guide disease control strategies requires accessible bioinformatics tools, ML models and researchers trained to interpret these data. Additionally, improving the accuracy of plant disease assessments by adopting advanced methods, including multispectral and chlorophyll fluorescence imaging, facilitated by recent advancements in artificial intelligence is required (Bock et al. 2022). To improve legume disease resistance prediction, it is important to capture, label and connect large amounts of clean data from various sources including plant breeding companies and research institutions. This will support the training of advanced, complex ML models in agriculture (Bayer and Edwards 2021). Interdisciplinary collaborative efforts for sharing data following FAIR principles such as findable, accessible, interoperable and reuse will accelerate progress in this field (Wilkinson et al. 2016).

Translating research findings into practical breeding programmes requires user-friendly tools tailored for legume breeders. These tools would help to incorporate ML predictions into their decision-making process, and support the challenges of legume breeding, such as balancing disease resistance with yield. Furthermore, emerging techniques including single-cell and spatial omics techniques could improve prediction accuracy in legumes, as it provides a clearer picture of changes during legume-pathogen interactions. Similarly, deep learning technologies such as large language models have the potential to generate high impact questions influencing legume-pathogen interactions and complement researchers’ investigations (Agathokleous et al. 2024). By addressing these challenges and using advanced computational approaches we can harness the full potential of multi-omics data, to provide more accurate prediction, which will ultimately aid in developing disease-resistant legumes using sustainable practices to meet global food security. 

## Conclusions

Multi-omics approaches provide insights into the different biological layers including genes, transcripts, proteins, and metabolites associated with complex traits such as disease resistance. These approaches are important for legumes, in which a narrow genetic diversity restricts the sources of variation available for resistance breeding. As pathogen evolution breaks down plant disease resistance, breeders require greater genetic diversity in their breeding pools to develop disease-resistant varieties. Legumes play an important role in meeting global food security, and integrating multi-omics with machine learning has the potential to decipher complex molecular mechanisms associated with disease resistance, improving prediction accuracy, supporting faster breeding cycles, and enabling more targeted gene editing.
